# Exploring the Ni-kshay SETU e-Platform Ecosystem: A Mixed-Methods Study on Strengthening Capacity Building Training for TB Care and Prevention (STEP) Among Interns

**DOI:** 10.7759/cureus.86092

**Published:** 2025-06-15

**Authors:** Shashikala Narayanappa, Harsh Shah, Balu PS, Jay Patel, Sujith H, Deepak Saxena

**Affiliations:** 1 Department of Community Medicine, Subbaiah Institute of Medical Sciences, Shivamogga, IND; 2 Department of Public Health Sciences, Indian Institute of Public Health, Gandhinagar, Gandhinagar, IND; 3 Center for AI Research and E-Health, Subbaiah Research Institute, Shivamogga, IND; 4 Department of Pulmonary Medicine, Subbaiah Institute of Medical Sciences, Shivamogga, IND

**Keywords:** capacity building, digital technologies, e-learning, medical interns, ni-kshay setu, tuberculosis

## Abstract

Introduction

Tuberculosis (TB) remains a critical global health challenge, particularly in low- and middle-income countries. In 2023, India had a large number of the world's TB cases, underscoring the urgent need for improved and effective training of healthcare providers involved in TB care and management. The World Health Organization (WHO) End TB Strategy emphasizes innovative approaches to enhance TB management, which include the use of digital tools for capacity building.

Objective

This study aims to evaluate the effectiveness of the Ni-kshay SETU (Indian Institute of Public Health, Gandhinagar, Gandhinagar, India) e-Platform in improving TB-related knowledge and practical skills among interns and to determine the scalability of the Ni-kshay SETU in training healthcare professionals.

Methodology

A mixed-methods approach was adopted, effectiveness was quantitatively assessed through pre- and post-test scores, feedback regarding the application was assessed for utility and interphase ease, and recommendation was assessed qualitatively. The overall performance of the digital e-learning platform was evaluated based on the WHO toolkit to assess the digital interface for learning among healthcare professionals.

Results

The study included 184 medical interns, with 156 registering on the Ni-kshay SETU platform. The pre- and post-test showed a significant increase in average scores from 14.49 (±6.67) to 20.46 (±7.84) post-training (p<0.001). Nearly two-thirds of medical interns felt that the training met their expectations, reported improvements in practical skills and critical thinking, and agreed to recommend it to their peers.

Conclusion

The Ni-kshay SETU e-Platform effectively improved TB care knowledge and skills among medical interns. Despite some challenges, the platform demonstrates significant potential as a sustainable training tool in TB management. Continuous updates and improvements in usability are essential for maximizing engagement and effectiveness in the long term.

## Introduction

Tuberculosis (TB) remains one of the leading infectious diseases globally, posing a significant public health challenge. In 2023, the World Health Organization (WHO) reported an estimated 10.8 million cases of TB globally, resulting in 1.25 million deaths, with the majority of the burden occurring in low- and middle-income countries where healthcare resources are limited [1]. India accounted for approximately one-fourth of the global burden of TB cases, and the challenges in the proper implementation of the program are mainly related to timely diagnosis and the initiation of treatment, along with a high prevalence of drug-resistant TB, which complicates treatment and control efforts [1].

The WHO End TB Strategy emphasizes the need for intensified research and innovation, focusing on the development of new tools, interventions, and strategies to optimize implementation and its impact on TB burden [2]. These efforts should address the major challenges in TB management, such as delayed care-seeking behavior, inaccurate diagnosis, delayed treatment initiation, and the limited resources and knowledge gaps among healthcare providers, especially in high-burden, low-resource settings [3-5].

With the rapid expansion of the National Tuberculosis Elimination Program (NTEP) and the continuous introduction of new guidelines, effective and scalable training strategies have become essential to keep healthcare workers updated on the latest developments in TB care [4,6]. The National Strategic Plan (NSP) 2017-2025 advocates for scalable systems and the adoption of e-learning methodologies for the capacity building of healthcare professionals to strengthen healthcare ecosystems [7]. Therefore, it is essential to develop quick reference and decision-support tools that are easily accessible, addressing the complexities encountered during the implementation of NTEP [8]. However, barriers such as poor management, inadequate infrastructure, and a lack of incentives often limit the program's ability to deliver long-term, systematic, and timely TB training to healthcare providers [9].

Digital tools such as mobile and web applications offer promising solutions for professional development by facilitating knowledge sharing, supporting timely care, and reinforcing adherence to clinical protocols [10]. For instance, interviews with healthcare providers revealed challenges, such as inadequate training, communication barriers, and resource constraints, which were further confirmed by a knowledge assessment of healthcare providers in Gujarat, India. This assessment indicated varied knowledge levels across healthcare provider cadres, with gaps noted among pharmacists and medical officers (MOs), while ASHAs and CHOs/Ayush MOs performed relatively better [6]. Studies, such as those conducted in China, have demonstrated that digital training platforms such as e-learning can effectively address training gaps by providing high-quality, accessible educational resources and achieving substantial certification rates among participants [11]. However, integrating these digital tools into real-world healthcare systems remains challenging, with factors such as local context, usability, and scalability often affecting their effectiveness [12,13].

Ni-kshay SETU (Indian Institute of Public Health, Gandhinagar, Gandhinagar, India) is a mobile application designed as a ready reckoner for healthcare providers managing TB under the NTEP [4]. Built through a participatory approach, it offers evidence-based, cadre-specific training in local languages to improve care-seeking, diagnosis, and treatment initiation [14]. Key features include regularly updated guidelines, multi-language support, an algorithm-driven resource library for decision-making, customizable content, an artificial intelligence (AI)-powered chatbot, and secure user authentication to ensure data confidentiality and accessibility even in remote areas [14]. This study aims to assess Ni-kshay SETU's impact on enhancing knowledge and practical skills among interns, thereby addressing the need for accessible, scalable training solutions within India's TB control efforts. By evaluating both the knowledge gains and user experience associated with Ni-kshay SETU, the study also aims to provide insights into the platform's potential as a sustainable training tool for capacity building on TB care among healthcare providers from all sectors (public and private).

## Materials and methods

This study employed a mixed-methods approach to evaluate the Ni-kshay SETU digital platform's effectiveness in enhancing tuberculosis (TB)-related knowledge and skills among interns.

Quantitative data

The assessments were conducted using standardized questionnaires to measure the knowledge level and skill development at the completion of all the training modules. The application usage pattern was monitored by the technical team, i.e., the number of times each medical intern accessed the specific module.

Qualitative data

A pre-structured questionnaire was used, which contained open-ended, three-point scale and rating scale (1-10) questions to assess expectation, perception, need, whether it could be recommended, any problems encountered, what they appreciated the most, and their suggestion to improve the application's usability. Only those interns who completed the post-test evaluation were included to provide their feedback on the course.

Study population and sampling

The training program targeted all medical interns enrolled in the Compulsory Rotatory Internship Program (CRIP) during the study period. A universal sampling strategy was employed to ensure the inclusion of all the interns available at that time. All participants were clearly informed about the study's objectives, and informed consent was obtained before participation. For the qualitative component, purposive sampling was used to gather feedback from interns who completed the course and took the post-test at the end.

Training program

The Ni-kshay SETU application was developed by a panel of experts from the Department of Public Health Sciences of the Indian Institute of Public Health, Gandhinagar, Department of Health and Family Welfare, Government of Gujarat, Gandhinagar, and subject experts on the National TB Elimination Program. Ni-kshay SETU is a concise, easily accessible learning material based on evidence-based practices, established guidelines, cadre-specific modules in local languages, and promoting ease of comprehension and applicability [4]. The Indian Institute of Public Health, Gandhinagar, approached us to conduct an exploratory study among interns for providing scalable training solutions and insights into the platform's potential as a sustainable tool for capacity building among healthcare providers.

The Ni-kshay SETU platform was designed to provide structured training on TB care and management through various modules. The training was organized into the following stages.

Orientation Session

Interns attended an introductory session on the Ni-kshay SETU platform. This session covered the purpose of the training, the benefits of using a digital platform for TB care, and a detailed explanation of the platform's features and navigation provided by the team of the Indian Institute of Public Health, Gandhinagar.

Registration and Pre-test

All interns were registered on the Ni-kshay SETU platform and completed a pre-test to assess their baseline knowledge of TB care. The pre-test consisted of a set of questions covering key areas of TB management, including diagnosis, treatment, and programmatic guidelines.

Training Modules

Over a four-day period, interns engaged with a series of digital training modules on the Ni-kshay SETU application, covering essential topics in TB care. Each module provided interactive ready reckoner content and scenarios designed to simulate real-world challenges in TB management, thereby allowing interns to apply their learning in a controlled environment.

Post-training Access

After the formal training period, interns retained access to all modules on the Ni-kshay SETU platform for ongoing reference and self-study. This accessibility was intended to support their continuous learning as they transitioned into real-world TB care roles.

Post-test evaluation and knowledge assessment

Following the training, interns completed a post-test assessment using a Ni-kshay SETU-administered question bank. This test assessed their knowledge gains across core areas, using a randomized order of questions to prevent response bias. A passing benchmark was set at 60% for the post-test, with additional categorization based on higher achievement levels (100%, 81%-90%, and 61%-80%).

The post-test included 25 validated questions, focusing on essential areas such as TB case definition, screening and diagnosis, treatment protocols, and preventive care. This tool underwent pre-validation with medical staff to ensure clarity, relevance, and accuracy.

Application usage monitoring

Additionally, the interns' interactions with the platform were monitored, tracking the frequency of module access, engagement with key resources, and the use of the platform's search and assistance features. This engagement data was analyzed to gain insights into usage patterns and assess the application's practical value as a learning tool.

Qualitative data: Feedback on the course

After completing the post-test, interns were invited to participate in a feedback survey to share their views on the Ni-kshay SETU platform, including its usefulness, the ease of use, expected benefits, impact on knowledge and skills, time required, and the modules they found most valuable. The survey also sought suggestions for enhancing the platform and included questions about the likelihood of recommending the platform to peers and suggestions for improving the application's performance.

WHO Reach, Effectiveness, Adoption, Implementation, and Maintenance (RE-AIM) framework

The WHO RE-AIM framework provides a toolkit for evaluating the implementation and scaling of digital innovations in tuberculosis. The usability and effectiveness of the innovations in real-world settings need to be understood for scaling up and framing policies, especially in resource-limited settings where health system constraints and accessibility are a matter of concern. The digital interventions in capacity building will help address the delay in diagnosis and treatment initiation and address the needs of a patient during the course of the treatment. This toolkit focuses on addressing the reach, effectiveness, adoption, implementation, and maintenance of the innovation in the world setting [15].

Data analysis

Quantitative data, including pre- and post-test scores, was analyzed using descriptive statistics to calculate means and percentages. A paired t-test was conducted to assess the statistical significance of knowledge improvement from pre-test to post-test scores. Usage patterns of the Ni-kshay SETU application modules were analyzed, categorizing users by the frequency of access to identify correlations with post-test performance.

Qualitative data from the feedback was analyzed thematically, identifying common themes in user satisfaction, perceived ease of use, and suggested improvements. The feedback was used to further interpret quantitative results and provide a comprehensive evaluation of the platform's effectiveness. The data was represented as frequencies, percentages in tables, and graphs. The qualitative data from the feedback and quantitative data from the assessment (pre- and post-test scores), along with the platform usage pattern, were integrated with the WHO RE-AIM framework to evaluate its usability and scaling up. The components of the WHO Reach, Effectiveness, Adoption, Implementation, and Maintenance (RE-AIM) framework were assessed based on the test scores, feedback, and number of times they accessed the various modules on the platform.

Ethical considerations

This study was conducted following ethical guidelines for research involving human participants. Interns were informed of the study objectives and provided with the option to participate voluntarily with consent. The confidentiality of all participants was maintained, and their data was anonymized for analysis. The Institutional Ethical Committee of Subbaiah Institute of Medical Sciences (IEC-SUIMS) issued approval IEC-SUIMS/12/2024-25.

## Results

A total of 184 medical interns enrolled for the internship program were invited to participate in the Ni-kshay SETU training study. Out of these, 156 interns (84.8%) registered for the training, and among the registered, 149 interns (95.5%) participated in the post-test evaluation. However, only 127 (81.4%) completed both the pre-test and post-test assessments; nearly 29 (18.5%) could not participate in the pre-test or post-test due to the technical glitches encountered during the assessment.

Nearly 156 medical interns on induction into the study demonstrated a moderate level of TB knowledge, with an average pretest score of 14.49±6.67 out of 25. The majority, 88 interns (56.4%), scored between 60% and 90%, while 38 (24.4%) scored below 60%. Following the training on the Ni-kshay SETU application, the average score increased significantly to 20.46±7.84 out of 25. Notably, 100 interns (64.1%) achieved scores above 90%, indicating substantial improvement in TB-related knowledge; about 49 (31.4%) scored between 60% and 90%, while seven (4.5%) could not take the assessment (Table 1).

**Table 1:** Distribution of the study participants according to their scores in the pre- and post-test. Data in parentheses is percentages.

Variables	Mean Score and SD	>90%, n (%)	60%-90%, n (%)	<60%, n (%)	Not attended, n (%)	Total, N (%)
Pre-test	14.49±6.67	8 (5.1)	88 (56.4)	38 (24.4)	22 (14.1)	156
Post-test	20.46±7.84	100 (64.1)	49 (31.4)	-	7(4.5)	156

Comparing the interns' performance based on their post-test scores, a paired t-test was conducted to evaluate the differences between pre-test and post-test scores among interns who scored above 90% and those who scored below 90% in the post-test. Only 127 (81.4%) medical interns undertook both pre- and post-test assessment. The analysis revealed no significant difference in pre-test scores between the two groups, indicating similar baseline knowledge levels. However, the post-test scores showed a statistically significant improvement (p<0.001) in the group scoring above 90%, demonstrating that the Ni-kshay SETU training effectively improved interns' knowledge in TB diagnosis and treatment (Table 2).

**Table 2:** Comparing the pre- and post-test scores using independent t-test (n=127).

Test scores	Post-test, >90% (n=100)	Post-test, 60%-90% (n=27)	P value
Pre-test scores	14.37±7.08	14.24±6.82	0.924
Post-test scores	24.17±0.95	20.92±1.72	0.000

Engagement and usage patterns on Ni-kshay SETU platform (n=156)

Nearly 156 interns accessed the training modules at various frequencies, with high engagement observed in key modules such as case definitions, 124 (79.5%); diagnostic algorithms, 108 (67.5%); treatment guidelines, 96 (61.5%); and adverse drug reactions (ADR), 92 (57.5%). Supplementary resources such as NTEP office orders, 86 (55.1%); TB preventive therapy, 78 (48.8%); differentiated care model, 88 (57.5%); and referral health facility, 120 (76.9%), were not accessed at all. Notably, interns who frequently engaged with these core modules demonstrated higher post-test scores, underscoring the effectiveness of repeated exposure to key content in reinforcing TB-related knowledge (Table 3).

**Table 3:** Distribution of participants according to the number of times they accessed various contents of the Ni-kshay SETU application (N=156), providing insights into engagement levels. Figures in parentheses are percentages. NTEP, National Tuberculosis Elimination Program; TB, tuberculosis

Modules in Ni-kshay SETU Application	Not Accessed Even Once	Accessed 1-10 Times	Accessed >10 Times
Case definition	32 (20.5)	112 (71.7)	12 (7.7)
Chat keyword	26 (16.6)	83 (53.2)	47 (30.1)
Chat question	155 (99.4)	1 (0.6)	-
Diagnostic algorithm	48 (32.5)	92 (57.5)	16 (10.0)
Differentiated care module	88 (57.5)	68 (42.5)	-
Adverse drug reaction	64 (42.5)	91 (56.9)	1 (0.6)
TB preventive treatment	78 (48.8)	82 (51.2)	-
Referral health facility	120 (76.9)	34 (21.7)	2 (1.3)
Treatment algorithm	60 (38.5)	93 (59.6)	3 (1.9)
Screening tool	65 (41.6)	89 (57.1)	2 (1.3)
Supplementary resource material			
Resource documents	81 (51.9)	74 (47.4)	1 (0.6)
NTEP guidelines	62 (39.7)	90 (57.7)	4 (2.6)
Office orders	86 (55.1)	70 (44.8)	-
Presentations	71 (45.5)	85 (54.5)	-
Videos	60 (38.4)	91 (58.3)	5 (3.2)
Resource material, others	62 (39.7)	29 (18.5)	66 (42.3)
Master search	131 (83.9)	15 (9.6)	10 (6.4)

Platform engagement and post-test scores

Intern engagement with the Ni-kshay SETU platform revealed a significant association between module access frequency and post-test performance. Interns who accessed core modules, such as case definitions, diagnostic algorithms, differentiated care, tuberculosis preventive therapy, and screening, were more likely to achieve post-test scores exceeding 90% (p<0.05). Furthermore, frequent engagement with modules on adverse drug reactions, treatment algorithms, NTEP guidelines, office orders, and presentations was associated with high post-test scores, demonstrating a strong statistical significance (p=0.001-0.004). These findings suggest that regular interaction with both core and supplementary learning materials enhances knowledge retention and reinforces learning outcomes (Table 4).

**Table 4:** Pattern of accessing the modules and resource materials among top scores and non-top scores in the post-test (n=149). ^*^The difference in the scoring among the interns as a result of the increased use of the modules significantly improved their performance. ^#^Most of the participants did not access these components at all during the course. ^a^ADR module was accessed more than 11 times by most of the participants. NTEP, National Tuberculosis Elimination Program; TB, tuberculosis; ADR, adverse drug reactions

Modules and Resource Material Accessed 1-10 Times	Top Scorer (n=100)	Non-top Scorer (n=49)	Total (N=149)	P value
Case definition	76 (71.0)	31 (29.0)	107 (71.8)	0.018^*^
Chat keyword	50 (64.1)	28 (35.9)	78 (52.3)	0.052
Chat question (not accessed)^#^	99 (66.9)	49 (33.1)	148 (99.3)	0.482
Diagnostic algorithm	67 (75.3)	22 (24.7)	89 (59.7)	0.011^*^
Differentiated care module	47 (58.0)	34 (42.0)	81 (54.4)	0.01^*^
Adverse drug reaction (>11 times)^a^	68 (76.4)	21 (23.6)	89 (59.7)	0.003^*^
TB preventive treatment	61 (76.3)	19 (23.7)	80 (53.7)	0.011^*^
Referral health facility (not accessed)^#^	73 (64.6)	40 (35.4)	113 (75.8)	0.33
Treatment algorithm	71 (78.0)	20 (22.0)	91 (61.1)	0.001^*^
Screening tool	64 (73.6)	23 (26.4)	87 (58.4)	0.033^*^
Resource documents	56 (76.7)	17 (23.3)	73 (49.0)	0.034^*^
NTEP guidelines	67 (76.1)	21 (23.9)	88 (59.1)	0.002^*^
Office orders	55 (80.9)	13 (19.1)	68 (45.6)	0.001^*^
Presentations	64 (77.1)	19 (22.9)	83 (55.7)	0.004^*^
Videos	67 (75.3)	22 (24.7)	89 (59.7)	0.016^*^
Resource material, others	38 (66.7)	19 (33.3)	57 (38.3)	0.007^*^
Master search (not accessed)^#^	85 (68.5)	39 (31.5)	124 (83.2)	0.274

Qualitative feedback on platform experience

A total of 111 interns (71.2%) responded to the post-training feedback survey. The majority found the Ni-kshay SETU platform useful and relevant to their learning needs. About 86 interns (77.4%) reported that the training met their expectations, and over 89 (79%) noted improvements in their practical skills and critical thinking abilities. While overall feedback was positive, some interns reported technical challenges, including difficulties with navigation and occasional connectivity issues. On average, interns spent approximately 4.88 hours completing the course (Table 5).

**Table 5:** Distribution of the study participants according to their opinion of the Ni-kshay SETU application as a teaching tool (n=111). Figures in parentheses are percentages.

Questions	Agree, n (%)	Neutral, n (%)	Disagree, n (%)
The course has met my expectations.	79 (71.1)	29 (26.1)	3 (2.7)
The course was practical with adequate knowledge, skills, and concepts on the subject that facilitated understanding.	86 (77.4)	22 (19.8)	3 (2.7)
The interactivity was suitable for the content.	81 (72.9)	27 (24.3)	3 (2.7)
The course was able to improve my critical thinking and ability to apply theory to practice.	86 (79.2)	19 (17.1)	6 (5.4)
Did you encounter any problems during the course? If yes, elaborate.	39(25.0)	-	72 (75.0)
The application is easy to access and use.	57 (51.3)	42 (37.8)	12 (10.8)
Was the amount of time it took to complete this course appropriate?	96 (86.4)	-	15 (13.5)
Rate your experience with the course.	Very good (9-10): 19 (17.1)	Good (6-8): 73 (65.7)	Bad (<5): 19 (17.1)
How likely are you to recommend this course to a colleague?	Very likely (9-10): 32 (28.8)	Likely (6-8): 53 (47.7)	Not likely (<5): 26 (23.4)
How much time did you spend on this e-learning course?	4.27±2.16 hours		

Suggestions for improvement included better navigation, more case-based learning, and expanded content on complex topics such as drug-resistant TB. When asked to rate the training experience, 82 interns (65.7%) gave it a score between 6 and 8 on a 10-point scale, while 19 (17.1%) rated it poorly. Despite the limitations, a majority of interns (76.5%) expressed willingness to recommend the platform to peers, indicating overall satisfaction with the training experience.

**Figure 1:**
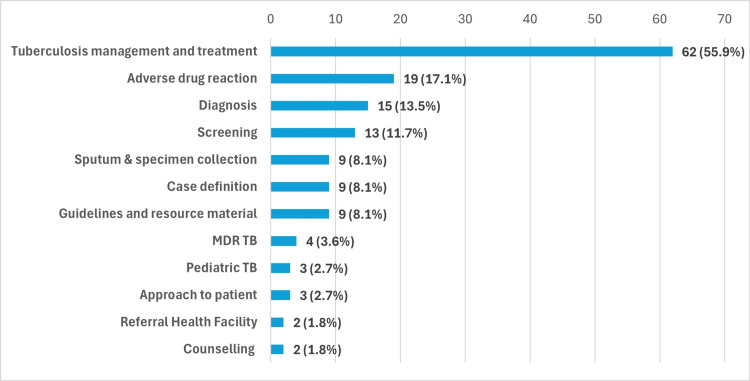
Distribution of the study participants according to the concepts they liked learning through the Ni-kshay SETU application (n=111). MDR, multidrug-resistant; TB, tuberculosis

The medical interns expressed that the module on TB treatment and diagnostic algorithms was the most appreciated, with 62 interns (55.8%) selecting it as their favorite. This was followed by 19 interns (17.1%) who preferred the adverse drug reaction (ADR) module, and 15 interns (13.2%) liked the screening module (Figure 1).

**Figure 2:**
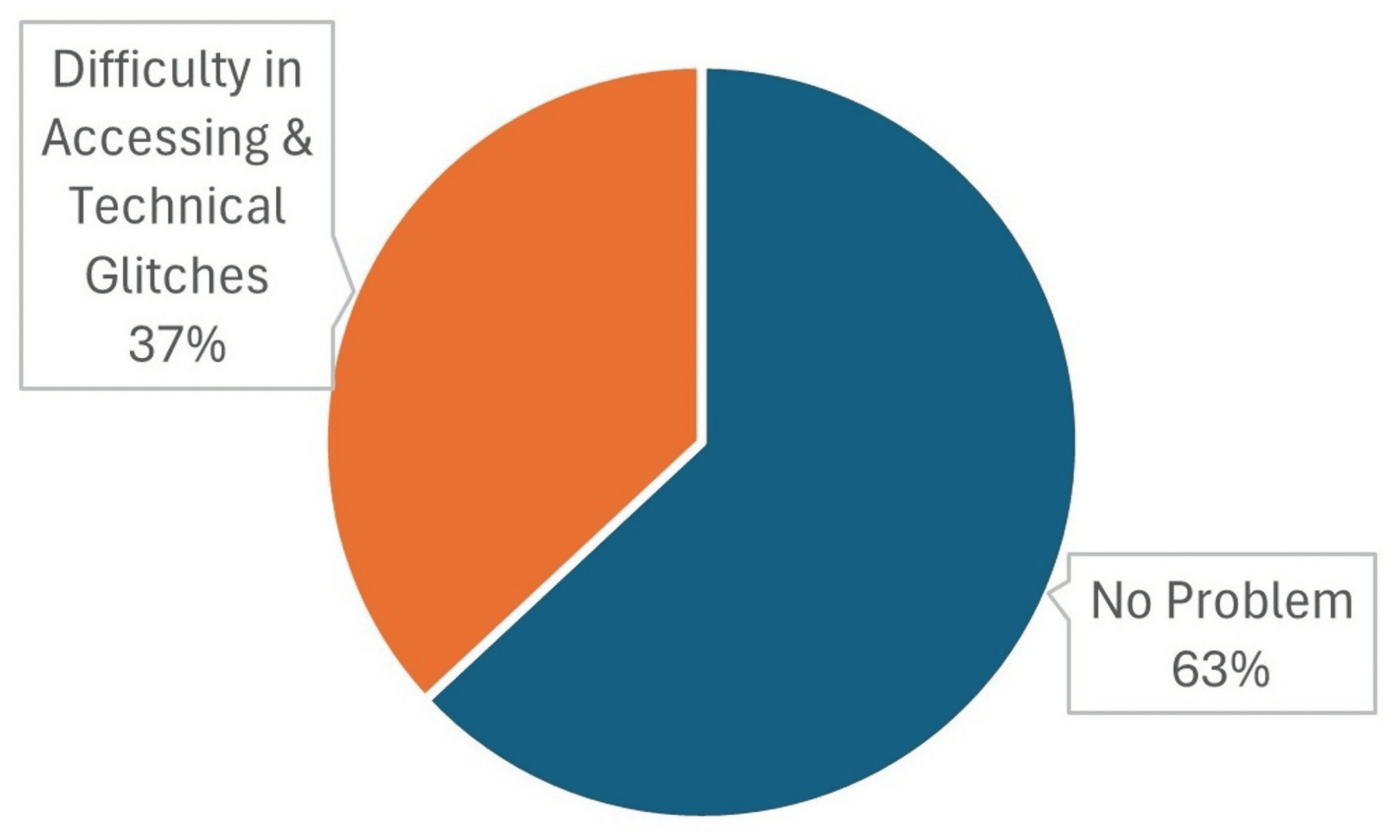
Distribution of the study participants according to the problems faced during the usage of the application (n=111).

About 70 (63%) interns did not face any difficulty during the course; however, nearly 41 (37%) of them faced certain difficulties (Figure 2).

**Figure 3:**
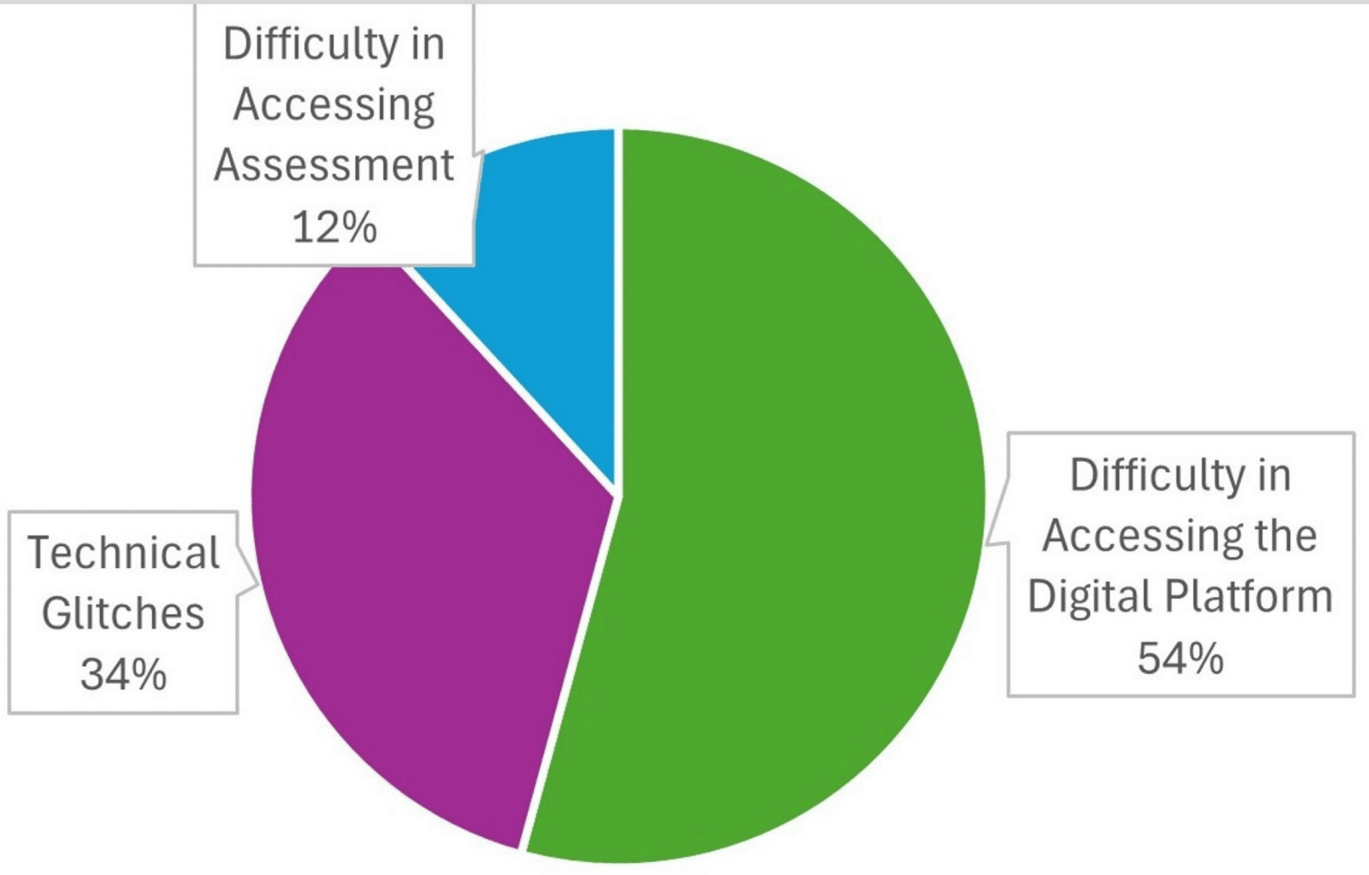
Distribution of the study participants according to the problems faced during the usage of the application (n=41).

Around 22 interns (54.0%) reported difficulties in accessing the digital platform. Of these, 14 interns (34.0%) encountered technical glitches that limited their ability to access training modules. Additionally, five interns (12.0%) faced issues accessing assignments or completing assessments (Figure 3).

WHO RE-AIM framework

The effectiveness of the Ni-kshay SETU digital training platform, developed by the Indian Institute of Public Health, Gandhinagar, has been evaluated using the WHO-endorsed RE-AIM framework. This framework assesses five key dimensions: reach, effectiveness, adoption, implementation, and maintenance, to determine the public health impact of interventions. The evaluation of Ni-kshay SETU across these dimensions is summarized below.

Reach

Nearly 85% successfully registered, and 81% completed the post-test assessment, reflecting a high participation rate, indicating that the platform was largely accessible and well-suited to the interns' schedules and learning needs. Despite this, one-third of the interns reported experiencing technical issues, particularly with navigation and connectivity. On the other hand, 57 interns (51.3%) provided positive feedback, noting that the application was easy to use. These findings highlight the importance of addressing technical barriers and enhancing the user interface to further improve accessibility and engagement.

Effectiveness

The Ni-kshay SETU platform was effective in enhancing TB-related knowledge among medical interns. The average pre-test score and post-test scores were significantly improved, with nearly 100 (67.1%) scoring above 90% post-training, compared to only eight interns (5.4%) before the training. Nearly 86 interns (77.4%) reported improvements in critical thinking and practical skills, while 79 interns (71.1%) felt that the course met their expectations. Additionally, the platform was cost-effective by overcoming the challenges and reducing the expenses related to travel, logistics, and resource allocation, making the training more accessible (access training modules at their convenience) and scalable across various healthcare provider cadres (public and private sectors).

Adoption

High participation, completion, satisfaction, and recommendation rates reflect the platform's effective design and content; flexibility to learn at one's own pace is a vital aspect that can be attributed to the high level of engagement. Medical interns spent an average of 4-6 hours to complete the course. Despite the overall positive reception, about one-third of users reported technical and accessibility challenges, though nearly half found the application easy to navigate. Addressing these usability issues will be important for improving the user experience and sustaining high adoption rates. However, the platform's successful integration will depend upon the support and favorable infrastructure in medical colleges, including reliable internet access, the availability of digital devices, and high levels of digital literacy among interns, which provides an enabling environment for early adoption.

Implementation

The Ni-kshay SETU platform supports effective tracking of training progress among intended beneficiaries, allowing for the ongoing monitoring of engagement and module completion. However, the addition of randomized assessment questions at the end of each module could further strengthen the evaluation of learning outcomes and help maintain participant engagement throughout the course. The successful implementation and potential for scaling up the platform are influenced by the involvement of stakeholders and policymakers. Environments that foster digital learning, through institutional support and policies promoting digital capacity building for healthcare providers, are especially conducive to wider adoption and sustainability of the platform.

Maintenance

Nearly two-thirds of the interns recommended the Ni-kshay SETU platform for their colleagues; thus, it has the potential for long-term usage, making it a sustainable and cost-effective training tool for healthcare providers from various settings. The platform's design includes built-in mechanisms for regular content updates, ensuring alignment with evolving TB care guidelines issued from time to time. Modules on TB management and adverse drug reactions (ADR) were particularly valued, emphasizing the need to keep such content updated and relevant. Addressing usability issues such as technical glitches, connectivity, and accessibility highlights the need for improvements in user interface and navigation to further enhance the effectiveness of the training application. Regular technical support, combined with platform enhancements such as randomized assessment question pools, case studies, and interactive learning scenarios, could further enrich the learning experience, catering to the diverse learning preferences, and improve overall user satisfaction and retention.

The evaluation of the Ni-kshay SETU platform among medical interns demonstrated high engagement with significant improvements in knowledge and critical thinking among the interns. Flexible self-paced learning, regular updates, and reduced logistics burden make it cost-effective. While usability challenges affected some of the users, addressing these technical issues, updating content regularly, and enhancing interactive features will support long-term engagement. The platform was generally well-received and found to be cost-effective and a feasible alternative considering the current scenario of the healthcare services and limitations of resources.

## Discussion

India's healthcare system is diverse and complex, comprising public and private sectors, rural and urban services, traditional healers, and providers from the Indian systems of medicine. This diversity, along with disparities in the availability of skilled manpower, is a major challenge in delivering consistent and high-quality tuberculosis (TB) care. Digital technology can help bridge these gaps by providing standardized knowledge and training, which can disseminate the knowledge in an efficient and timely manner to improve the quality of TB care [16-18]. Digital technologies offer solutions to challenges such as inadequate training, communication barriers, and resource constraints hindering progress toward the End TB targets. Therefore, leveraging digital technologies for e-learning initiatives is effective and efficient for knowledge and skill development among healthcare professionals, especially in low- and middle-income countries [19,20].

The present study assessed the effectiveness of the Ni-kshay SETU e-learning platform in enhancing tuberculosis (TB) knowledge and skills among medical interns through a mixed-methods approach. The findings revealed a significant improvement in interns' knowledge, with average test scores increasing from 14.49 pre-training to 20.46 post-training. Additionally, two-thirds of the participants reported that the course met their expectations and expressed willingness to recommend it to their peers. These results contribute to the growing evidence supporting the use of digital tools as innovative training solutions to address the challenges in TB care, especially in resource-constrained environments, where traditional training methods are challenging to implement [2,21-23].

High engagement rates observed in specific training modules, such as case definitions and diagnostic algorithms, underscore the importance of targeted content in enhancing learning outcomes. This is consistent with previous studies that have shown that tailored digital training can effectively close knowledge gaps among healthcare providers [24]. Moreover, the significant association between module access frequency and post-test scores suggests that repeated exposure to educational materials reinforces knowledge retention, a concept supported by the principles of spaced learning [25]. These findings emphasize the need for investment in digital education strategies that address current knowledge deficiencies and adapt to the evolving prospect of tuberculosis care and management.

The incorporation of case studies and interactive scenarios reflects practical, real-world situations that require the application of the knowledge gained. This is consistent with educational theories that advocate for experiential learning as a means to deepen the understanding and application of complex concepts [26]. The interactive elements in the Ni-kshay SETU platform could promote active learning by enhancing critical thinking and problem-solving skills among the young professionals [27].

The feedback collected from interns indicated that the platform met their expectations from the course and they expressed willingness to recommend it to peers, highlighting its relevance and user satisfaction. Despite the overall success of the Ni-kshay SETU platform, the study identified some technical challenges as barriers to usability. Approximately 35% of interns reported difficulties with navigation and connectivity, and only about half of them found ease with navigation, which could hinder the platform's adoption and effectiveness in diverse healthcare settings. This finding is similar to studies that highlight the importance of user-friendly interfaces and reliable internet access in the successful implementation of e-learning platforms [28]. Addressing these technical issues is crucial for maximizing the platform's reach and user experience to ensure that it serves as an effective training tool for healthcare providers at all levels.

Ni-kshay SETU e-Platform-based training can be adopted on a large scale and serve as a sustainable option for the Ni-kshay SETU e-Platform. Finally, the Ni-kshay SETU e-Platform provides a promising tool for enhancing TB care and ensuring sustained engagement and effective training of future healthcare providers in the fight against tuberculosis. The study suggests that logistical barriers could be overcome by the institutional/organizational support for e-learning, which can facilitate broader access to TB training by all healthcare professionals at all levels.

Public health and policy implications

The Ni-kshay SETU e-Platform promises to be a scalable, adoptable, and sustainable solution for providing tuberculosis (TB) care training across India to address the knowledge gaps and resource limitations in the healthcare sector. The findings of this study highlight that digital innovations in healthcare training, in resource-limited settings, can be an important tool in the fight against TB. Promoting digital training, such as Ni-kshay SETU, into public health initiatives will ensure that healthcare providers receive timely, relevant training to improve TB care by addressing key challenges, such as delays in diagnosis and treatment, thus reducing the socioeconomic burden of TB on patients and communities. Supportive policies that increase access to digital tools and training resources will play a crucial role in achieving the goal of TB elimination across the nation.

Clinical implications

Timely and up-to-date training will empower clinicians to make informed decisions, minimize errors, and manage complex cases, including drug-resistant TB, and prevent adverse outcomes from tuberculosis for the patient and their families. With essential knowledge and skills, delays in diagnosis and treatment initiation can be reduced, ultimately improving TB care quality, benefiting the individuals and communities alike by reducing TB transmission and contributing toward achieving broader public health goals.

## Conclusions

The Indian healthcare system is highly diverse. However, the private sector often serves as the first point of care for many TB patients, which lacks standardized diagnostic and treatment protocols, leading to inconsistent care and potential delays in diagnosis, treatment initiation, and completion. Building healthcare provider capacity across both public and private sectors is essential to address the challenges in TB care. Digital ecosystems offer timely and effective training solutions, especially in resource-limited settings, enabling healthcare providers to acquire essential knowledge and skills. Starting this training early, such as during medical internships, can help close knowledge gaps and enhance the implementation of NTEP strategies. Combining e-learning with in-person orientations may strengthen training quality, sustainability, and effectiveness.

The Ni-kshay SETU e-Platform has the potential to be an effective and sustainable tool for capacity building in tuberculosis (TB) diagnosis, management, and care, among medical interns. This study found significant improvements in medical interns' knowledge and skills related to TB care and management, underscoring the effectiveness of digital training in addressing critical educational gaps within the healthcare system. Integrating digital health solutions can strengthen the healthcare workforce and foster global TB control initiatives. However, its effectiveness in widening its reach will depend on improving user satisfaction, sustaining long-term engagement depends on addressing technical issues, and enhancing usability will require ensuring regular content updates to keep information relevant over time.

## Appendices

Table 6 shows the Ni-kshay SETU training modules for interns.

**Table 6:** Ni-kshay SETU training modules for interns. TB, tuberculosis; ADR, adverse drug reactions; NTEP, National Tuberculosis Elimination Program; TPT, tuberculosis preventive treatment; DSTB, drug-sensitive tuberculosis; DRTB, drug-resistant tuberculosis; PwTB, person with TB

Modules	
Case finding	Screening tool
	Case definitions
	Active case findings
Manage TB, diagnostic care cascade	Sputum collection process
	Guidelines for sputum transportation
Manage TB, ADR	Guidance on adverse drug reactions
Manage TB, treatment care cascade	Components of counselling
	DSTB and DRTB treatment guide
	TB and nutrition
Manage TB, TPT	TB preventive treatment
Manage TB, differentiated care model	Differentiated care of PwTB
NTEP intervention	Post-treatment follow-up
	TB treatment adherence management
